# Mechanical Dentistry

**Published:** 1880-04

**Authors:** Edgar D. Swain


### ARTICLE IV.

### *Mechanical Dentistry.*

BY DR. EDGAR D. SWAIN.

The subject of Mechanical Dentistry, though occupying a back seat in the profession, is not entirely devoid of interest; and although minor improvements are being continually made and brought before the profession, they fail to elicit that interest which attaches to anything new in the so-called operative branch of our profession.

To be able to put six blocks of porcelain teeth upon a base of rubber or celluloid fully satisfies the average practitioner, and with the present indefinite ideas as to where the line of demarcation between the mechanical and operative branches should be drawn, it really seems strange that we have a division at all. With more experience and more intimate acquaintance with the duties devolving upon the practitioner of dentistry, I am inclined to the opinion that were the mechanics taken out of the science of dentistry, little would be left to the gentlemen who so loftily ignore the title of Mechanical Dentist.

To prepare the cavity of decay in a tooth for the reception of a filling of gold or other material may be operative or surgical, but with the packing of the first piece of filling again commences the mechanical; therefore, there is left to the operative or surgeon dentist the preparation of the cavity to receive the filling, and the treatment of the few diseased conditions of the soft tissues of the oral cavity which accidentally fall into our hands, as with the surgeon, whose duty, as such, ends with the dressing of the stump of the amputated limb, when the mechanic is called to provide the substitute.

The slaughter of the innocents, we are daily reminded, continues where the free use of gas prevails; and the yawning pocket of the *surgeon* dentist is always open to receive one dollar per tooth for extracting, and eight dollars, or less even, to supply the substitute which has been made necessary, through the incompetency of himself as a surgeon dentist. Having filled the teeth repeatedly, and with each failure assuring the suffering patient that her teeth "do decay away from the fillings so," the unfortunate, tired with both the expenditure of money and time, backed by the assurances of the "surgeon dentist," as blandly expressed as the innocence of the "Heathen Chinee," that the only thing left is the extraction of the ruined members, and the securing of one of his unequaled substitutes therefor; and from that time no more filling, no more pain, no more expense.

Since I last addressed this Society upon this subject, there have appeared several quite important improvements in this branch of our profession; and I think that, with the generally increasing intelligence of our brethren in the profession, there is a growing tendency to save or utilize partially decayed teeth and the fangs of all the teeth, even though the operations be not of the character properly denominated operative, or the building of contour fillings, but by placing upon them artificial crowns of either gold or porcelain. It is evident, from the numerous improvements upon old methods, and inventions of new, that our thinking men and inventors are giving more thought to this branch of our work than in former years, and that the results thus far warrant continued exertions.

Of these improvements or inventions I shall speak but briefly, and with the hope of increasing the interest of those present in this direction.

First, I will call attention to the methods of Dr. Richmond, of San Francisco, and Dr. Hale, of St. Louis, for constructing and setting gold crowns upon the fangs of the bicuspids and molar teeth—methods which, to my mind, are to mechanical dentistry what the rubber dam was to operative. Either of these methods contemplates the manufacture of the artificial crown out of the mouth and in the laboratory, except so much fitting as may be necessary to the fang they are to be worn upon.

The Richmond method is the most simple and easy of the two, and except in the artistic imitation of the form devised and used by the Creator, answers equally well the purpose.

The first part of the operation must be classed under the head of Dental Surgery or Medicine, as it consists in the curing of any diseased conditions which may exist in the soft tissues enveloping the fang, as formed abscess, ulceration, periostitis, etc. This accomplished, a band or tube of coin gold is, with the plate benders, pliers, or other convenient manner, fitted to the fang so closely as, when soldered, to require considerable force to send it to its place; this tube to extend below the margin of the gums, in some cases as far as the edge of the process, and as high as the contemplated crown. The metal being soft, it will be found to adapt itself readily to the tooth or fang to be fitted. This tube or barrel forms the sides of the crown, and when fitted should be cut a little shorter than the required length contemplated, then upon the end solder a disk of same material. Then melting down some of the clippings from the plate used, into beads, these to be slightly flattened under a hammer, so as not to roll about, are soldered upon the disk and filed into cusps, two to be used for a bicuspid tooth, and four, more or less, for a molar. The æsthetic taste of the operator will, of course, settle the question as to the finish. We now have a gold shell shaped more or less like the crown of a tooth. The solder used is very fine, and so alloyed as to retain the color of the coin gold, consequently no unsightly seems are seen in the finished work. Frequently these shells are placed over enough of the crown of the natural tooth to retain them firmly without the aid of screws; but this being entirely gone, screws of nickel or gold may be set in the root. Fill the shell with oxy-chloride of zinc, and force home to its place, and we have an artificial crown, without subjecting our patient to the all day's torture of packing enough gold foil to have built it up, and firmer and more durable than possible to make with the old method. The fine mechanical ingenuity, close workmanship of the operator, with nicety of adaptation, all add to the success of the operation.

Dr. Hale's method differs not in the result so much as in the manner of arriving at it. First, cast-iron dies are procured of the grinding surfaces of bicuspids and molars, and that part of the crown is swedged into a disk sufficiently large to form the crown contemplated, then V shaped notches are cut out, allowing the sides to be brought down, forming the sides when completed. This crown is drilled at some convenient place to allow the packing of amalgam, to retain it in place, screws having previously been set, about which the amalgam is to be packed. This, when completed, makes a very durable as well as artistic piece of work.

Both of these methods provide for the facing of the crown with porcelain, whenever desirable to hide the gold, as often is the case with bicuspids, by simply sawing out enough of the outer portion of the gold crown to allow a plate tooth to be secured there. This I have done in several cases with results highly satisfactory to the wearer.

The Richmond process also provides for the setting of pivot teeth upon fangs of the six anterior teeth, so constructed as to prevent the splitting of the fang. The process for accomplishing this is so complicated that I will not attempt to make it clear in an essay of this character. I will, however, say that I have not been entirely successful with this work, and acknowledge the fault to be in a measure owing to imperfect manipulation.

In regard to the use of oxy-chloride of zinc for securing the gold crowns in place, I desire to caution all that they be very particular to remove every vestige of it from about the neck of the tooth, going with properly shaped instruments under the free margin of the gum, also wash out repeatedly with water, or, what is better, bicarbonate of soda and water. Unless this is thoroughly done the parts will slough away, leaving an unsightly operation, as well as removing the valve formed by the gingival margin to keep the secretions from penetrating to the oxy-chloride inside the shell. I met with this experience in one case, and have since been cautioned by Dr. Richmond himself, who had experienced the same trouble.

Next I desire to call your attention to some improvements made by Dr. Holbrook, of Waukesha, Wis., consisting in changes made in the impressions and plaster casts, so as to secure results which every mechanical dentist has long felt the need of. It does away entirely with the usual form of vacuum cavity so long in use, securing a much better adaptation, and largely, if not entirely, avoiding the liability of rubber and celluloid plates to rock, as we express it, and decreasing very largely the liability to break through the mesial line of the plate, also securing to the lower plate a very good adherence from atmospheric pressure or suction. This is accomplished without the use of soft rubber edges, flanges, or any of those devices which have heretofore been tried and found wanting.

The improvements work equally well with all kinds of work, metal or plastic, and I feel confident that their adoption by those who manufacture the porcelain work would assist very materially in overcoming some of the greatest difficulties they have to contend with. The process through which these results are accomplished I am debarred from giving you by letters patent.

And, now, one more secret and I am through. All hear more or less complaint from patients wearing rubber plates, of a burning sensation, feverish, and often congestion of the parts under the plate. A small piece of sulphate of zinc, anywhere in the neighborhood of ten grains, dissolved in a large spoonful of vinegar, and washing the plate thoroughly with the preparation, has frequently resulted in the complete cure of very aggravated cases. This may not be a specific, but its use has been so satisfactory that I felt it worth giving to the profession; and if others give to their patients the same amount of relief which several of mine have received, your money and time spent in attending this session of our Association will not have been entirely wasted.—*Transactions of Illinois State Dental Society.*

